# *DRD1* and *DRD2* Receptor Polymorphisms: Genetic Neuromodulation of the Dopaminergic System as a Risk Factor for ASD, ADHD and ASD/ADHD Overlap

**DOI:** 10.3389/fnins.2021.705890

**Published:** 2021-09-29

**Authors:** Maria Addolorata Mariggiò, Roberto Palumbi, Angela Vinella, Riccardo Laterza, Maria Giuseppina Petruzzelli, Antonia Peschechera, Alessandra Gabellone, Ottavio Gentile, Alessandra Vincenti, Lucia Margari

**Affiliations:** ^1^Department of Biomedical Sciences and Human Oncology, University of Bari Aldo Moro, Bari, Italy; ^2^Pediatric Surgery Unit, Giovanni XXIII Hospital, Bari, Italy

**Keywords:** autism spectrum disorder, ADHD, ASD/ADHD overlap, dopaminergic system, dopamine receptors, polymorphisms, neuromodulation, neurobiology

## Abstract

The dopaminergic system (DS) is one of the most important neuromodulator systems involved in complex functions that are compromised in both autism spectrum disorder (ASD) and attention deficit/hyperactivity disorder (ADHD), conditions that frequently occur in overlap. This evidence suggests that both disorders might have common neurobiological pathways involving the DS. Therefore, the aim of this study was to examine the *DRD1* and *DRD2* dopamine receptor single nucleotide polymorphisms (SNPs) as potential risk factors for ASD, ADHD, and ASD/ADHD overlap. Genetic data were obtained from four groups: 75 ASD patients, 75 ADHD patients, 30 patients with ASD/ADHD overlap, and 75 healthy controls. All participants were between 2 and 17 years old. We compared the genotypic and allelic frequency of 18 SNPs among all of the study groups. Moreover, in the case of statistically significant differences, odds ratios (OR) were obtained to evaluate if the presence of SNPs might be a risk factor of developing a specific clinical phenotype. This study found that *DRD1* and *DRD2* receptors SNPs might be considered as potential risk factors for ASD and ADHD. However, only *DRD2-12* (*rs7131465*) was significantly associated with a higher risk for the ASD/ADHD overlap. These data support the hypothesis of the genetic neuromodulation of the DS in the neurobiology of these conditions.

## Introduction

Autism spectrum disorder (ASD) and attention deficit/hyperactivity disorder (ADHD), as well as bipolar disorder or schizophrenia, are neuropsychiatric disorders characterized by strong genetic bases (Sullivan et al., 2012; Woodbury-Smith and Scherer, 2018; Rylaarsdam and Guemez-Gamboa, 2019; Grimm et al., 2020). The dopaminergic system (DS) is involved in the regulation and the neuromodulation of some central nervous system (CNS) functions, such as social skills, the perception and the reward mechanisms for social activities, and attention and motor functions (Pavãl, 2017; Klein et al., 2019; Madadi Asl et al., 2019). Moreover, over the last two decades, several studies underlined that alterations in DS contribute to both ASD and ADHD (Iversen and Iversen, 2007; Cousins et al., 2009; Del Campo et al., 2011; Dichter et al., 2012; Owen et al., 2017).

These alterations may be related to different consequences: a selective deficit of dopamine (DA), and genetic mutations to the genes involved in synaptic homeostasis, as DA receptors, membrane transporters, or the enzymes designated to DA degradation or reuptake.

Genome-wide association studies (GWAS) significantly contributed to the identification of several genome variants known as single nucleotide polymorphisms (SNPs) associated with neuropsychiatric disorders (Cross-Disorder Group of the Psychiatric Genomics Consortium, 2013, 2019; Cross-Disorder Group of the Psychiatric Genomics Consortium, Lee et al., 2013). These genomic variations may remain silent, without functional implications. In other cases, SNPs can give rise to missense or non-sense mutations, gene expression, or splicing alterations. When a DS receptor region is involved, SNPs can cause increase or reduction, until the absence, of receptor protein. Alternatively, binding potential or binding affinity of receptor proteins for the ligand can also be modified (Sullivan et al., 2012; Cross-Disorder Group of the Psychiatric Genomics Consortium, Lee et al., 2013).

Given the multitude of gene variants and possible mechanisms, several studies investigated the correlation between the SNPs involving the DS and ASD or ADHD.

Attention deficit/hyperactivity disorder is a neurodevelopmental disorder (NDD) characterized by a persistent pattern ofattention deficit, hyperactivity, and impulsivity; it is one of the most common NDDs with a complex etiology and a strong genetic component (Nigg, 2013; Matthews et al., 2014; Demontis et al., 2019; Grimm et al., 2020). The clinical symptomatology of ADHD is linked to a series of alterations of functions regulated by the DS in the CNS. Furthermore, functional neuroimaging evidence has offered results about dopaminergic dysfunction in patients with ADHD, supporting the possible role of catecholaminergic dysregulation in the neurobiology of the disorder (Nigg, 2013).

As in ADHD, the DS is also involved in the ethology of ASD (Pavãl, 2017; Madadi Asl et al., 2019). ASD is a disorder characterized by two main core symptoms: a social communication and interaction deficit and the presence of repetitive and restricted interests and behaviors. Most of the functions disrupted in ASD are regulated by the DS. For example, the prefrontal cortex and the mesocorticolimbic circuit are both involved in executive functions and social cognition, while a nigro-striatal pathway alteration might explain the motor symptoms of ASD (Pavãl, 2017).

Recent studies have already identified hundreds of ASD-related gene variant encoding for synaptic proteins, transcription factors, epigenetic modulators and molecules involved in intracellular signaling (Castellanos and Tannock, 2002; Wise, 2004; Yin and Knowlton, 2006; Balleine et al., 2007; Hettinger et al., 2012). The DS plays a role in motor functions, reward and motivation which are altered in ASD. Patients with ASD display inappropriate social behavior (Mayes et al., 2011; Neale et al., 2012; Lamanna et al., 2017). Furthermore, some genetic studies have identified several SNPs or gene mutations related to the DS in patients with ASD (Craig et al., 2015, 2016).

Autism spectrum disorder and ADHD share common clinical features related to the impairment of several functions, such as attention skills, executive functions, and motor and social skills (American Psychiatric Association, 2013; Craig et al., 2015, 2016; Antshel and Russo, 2019; Gudmundsson et al., 2019). The overlap between ASD and ADHD is the clinical condition in which the two disorders are comorbid and the respective symptoms occur in the same patient. Since the publication of the fifth edition of the Diagnostic and Statistical Manual of Mental Disorders (DSM-5), ADHD is no longer an exclusion criteria for an ASD diagnosis and vice versa (American Psychiatric Association, 2013).

According to a recent review, the prevalence of ASD/ADHD overlap has increased over the years, and these disorders seem to share genetic heritability and some clinical features (Antshel and Russo, 2019). Other studies aimed to identify possible risk factors for these condition (Craig et al., 2015, 2016; Lamanna et al., 2017; Gudmundsson et al., 2019), but its neurobiology is still unclear.

Therefore, the purpose of this study was to provide new results that might confirm and support the involvement of DS in the pathogenesis of ASD, ADHD, and their overlap, focusing on dopaminergic receptor SNPs as possible genetic risk factors for these conditions.

## Materials and Methods

### Participants

For the study, patients diagnosed with ASD, ADHD, and ASD/ADHD overlap were recruited at the Childhood and Adolescence Neuropsychiatry Unit, University of Bari Aldo Moro, from 2015 to 2019.

The inclusion criteria were patients diagnosed with ASD, ADHD, and ASD/ADHD overlap, and aged between 2 and 17 years. The diagnoses were made according to the diagnostic criteria of the DSM-5 (American Psychiatric Association, 2013). We decided to consider ASD/ADHD overlap as an individual group in order to identify dopamine receptor SNPs as possible genetic risk factors of this distinct clinical disorder. The clinical diagnostic procedures included a full medical history interview, a neurological examination, and the administration of standardized protocols. We recruited 75 patients with ASD, 75 patients with ADHD and 30 patients with ASD/ADHD overlap. All patients included in the study were Caucasian.

The exclusion criteria were patients suffering from ASD and ADHD attributable to known genetic syndromes or other medical conditions (e.g., ASD-like symptoms might occur in fragile X syndrome; ADHD-like symptoms might be caused by drug intoxication or fetal alcohol syndrome).

For comparison and risk assessment of genotypes, 75 subjects aged between 2 and 17 years that had surgery and without any neurodevelopmental disorders were recruited at the Pediatric Surgery Unit, Giovanni XXIII Hospital, Bari, as controls.

The study was approved by the Local Ethical Committee (protocol number 592/12) and for all participants, informed consent was collected from their parents.

### Genotyping

The choice of polymorphisms was influenced by several factors. *DRD1* and some *DRD2* SNPs involved in this study were already known in the literature. Furthermore, using http://www.ncbi.nlm.nih.gov/nuccore/209977039?report=genbank&to=72685, we searched for all polymorphisms of the *DRD2* gene that are currently identified.

Since the methylation profiles of regions containing CpG islands could influence the levels of gene expression, using the CpGplot program of the EMBOSS package (available at https://www.ebi.ac.uk/Tools/seqstats/emboss_cpgplot/) we identified two regions within the introns of the *DRD2* gene that are unusually enriched with CpG dinucleotides; the first extends from nucleotide 4,634 up to nucleotide 5,660 (therefore longer than 1 kb), and the second extends from nucleotide 5,740 up to 5,953 (214 base pair long).

In these regions, we selected SNPs having an allelic frequency not less than 10% (0.1) in principle and, among these, only those that could be discriminated using the restriction fragment length polymorphism (RFLP) technique were considered.

This technique involves the use of restriction enzymes that recognize and cut specific DNA sequences. The enzymatic cutting is usually carried out in correspondence with the polymorphic sequence, allowing the recognition of the nucleotide variation.

The search for restriction enzymes to be used was conducted using the programs available on the New England Biolabs website^1^. The first program used was NEBcutter^®2^, which allows the identification of restriction enzymes able to discriminate the polymorphic sequence. We then moved on to the Primer3 program (see 0.4.0) (available at http://bioinfo.ut.ee/primer3-0.4.0/primer3/) to design amplification primers for the restriction sites and, finally, the REBsites program^3^ was used to predict the length of the fragments obtained after the restriction enzyme cutting. The genotyping of the recruited subjects was carried out using venous blood samples from patients and controls. To isolate the leukocytes of the study subjects, a sample was taken in tubes containing sodium citrate. A total of 10 ml of peripheral blood was mixed in a 1:1 ratio with Emagel (Piramal Healthcare, Northumberland, United Kingdom) heparinized (5 U.I. of heparin per ml of Emagel). The obtained solution was placed on a rotor for 10 min at the end of which the red cells were left to settle. The supernatant thus obtained was centrifuged at 1,600 rpm for 10 min. The pellet was re-suspended in 5 ml of 1X PBS and centrifuged at 1,600 rpm for 10 min. To remove the present cells, an osmotic shock was applied: the pellet was then re-suspended in 1 ml of 0.2% NaCl and vortexed for 1 min. Subsequently, 1 ml of 1.6% NaCl was added and the suspension was then centrifuged at 1,200 rpm for 10 min. Where necessary, the osmotic shock was repeated. The pellet was finally re-suspended in 1 ml of physiological solution and the leukocytes were counted in the Burker chamber. After cell counting, 10 × 10^6^ cell aliquots were used to extract DNA using DNAzol^®^ Reagent (Life Technologies, Carlsbad, CA, United States).

**FIGURE 1:**
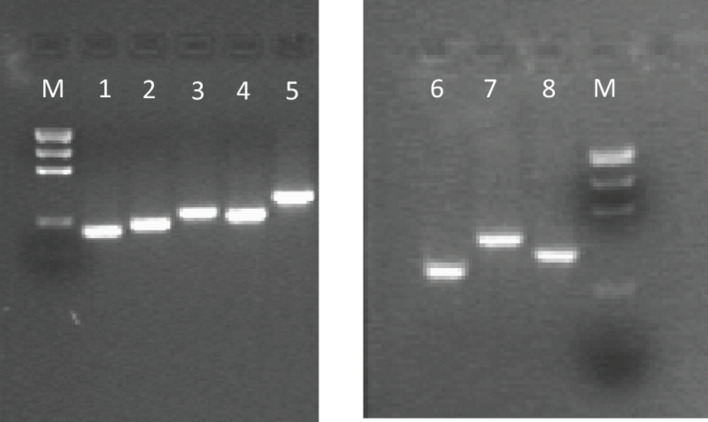
Example of gel electrophoresis pattern of PCR amplicons about some analyzed *DRD* SNPs with their size. M, Precision Molecular Mass Ruler, Bio-Rad Laboratories, Inc., (size of DNA fragments: 1000, 700, 500, 200, 100 bp). PCR amplicons: 1, *DRD 1-A* (166 bp); 2, *DRD 1-B* (187 bp); 3, *DRD 1-D* (232 bp); 4, *DRD 2-A* (213 bp); 5, *DRD 2-B* (293 bp); 6, *DRD 2-15* (250 bp); 7, *DRD 2-16* (362 bp); 8, *DRD 2-17* (300 bp).

The DNA concentration was measured by spectrophotometer and the solution was diluted with H2O RNasi and DNasi free (SIGMA) to obtain a final value of 100 ng/μl. Each polymorphic region was amplified using 100 ng DNA, 5 μl 10X PCR buffer, 3 μl 25 mM MgCl2, 2 μl 10 mM dNTPs mix, 0.5 μl AmpliTaq Gold 5 U/μl (Life Technologies, Carlsbad, CA, United States) and 1 μl of specific primer (IDT Inc., Coralville, IA, United States). The thermal protocol used was the same for all reactions, with an annealing temperature of 57°C and several cycles equal to 40. Table 1 shows the 18 polymorphisms selected for the study, their related gene and expected PCR amplicon size. Individual amplicons electrophoretic runs are displayed in Figure 1.

**TABLE 1:** List of analyzed polymorphisms of *DRD1* and *DRD2* genes and of the primer sequences with the expected amplicon size.

Gene (rsID)	SNP primer sequences	Expected amplicon size (bp)
*DRD 1-A (rs686)*	*FOR: 5*′*-GTGTGTTGGAAAGCAGCAGA-3*′ *REV: 5*′*-CCATCACACAAAACGGTCAG-3*′	*166*
*DRD 1-B (rs4532)*	*FOR: 5*′*-GGCAGAGGTGTTCAGAGTCC-3*′ *REV: 5*′*-CGGTCCTCTCATGGAATGTT-3*′	*187*
*DRD 1-C (rs265973)*	*FOR: 5*′*-GCATGCCAATTTGCTCTTG-3*′ *REV: 5*′*-GGATTAAAGAGGATCCAGTCCA-3*′	*100*
*DRD 1-D (rs265975)*	*FOR: 5*′*-CCTCTCATGTCCCTCTCCAA-3*′ *REV: 5*′*-GAGCAAGGACAACAGGAAGC-3*′	232
*DRD 2-A (rs1076560)*	*FOR:5*′*-GACAAGTTCCCAGGCATCAG-3*′ *REV:5*′*-GGCAGAACAGAAGTGGGGTA-3*′	213
*DRD 2-B (rs1800497)*	*FOR:5*′*- AAATTTCCATCTCGGCTCCT-3*′ *REV:5*′*-GAGGAGCACCTTCCTGAGTG-3*′	293
*DRD 2-C (rs1079597)*	*FOR: 5*′*-TTTCCCTTCTGTGGGATGAG-3*′ *REV: 5*′*-GGAGGTTGCAATAGGCAAGA-3*′	274
*DRD 2-E (rs7118900)*	*FOR: 5*′*- CGCAGTAGGAGAGGGCATAG-3*′ *REV: 5*′*-ATGGGAGCTTCAAAGGGAAG-3*′	*348*
*DRD 2-1 (rs144851051)*	*FOR: 5*′*- CTCAGCCTCCCAAGTAGCTG-3*′ *REV: 5*′*- GCTGTCCACATGCTGAAGAA-3*′	*346*
*DRD 2-2 (rs11608185)*	*FOR: 5*′*-GTGTGCATGGCTGTGTCC-3*′ *REV: 5*′*- GCTGCTGTGAGGGTTATATAGGA-3*′	396
*DRD 2-7 (rs35352421)*	*FOR: 5*′*-CCTGCACCCCAGATTCAG-3*′ *REV: 5*′*- CTGTTTCCTCTCTGCCAACC-3*′	375
*DRD 2-8 (rs2245805)*	*FOR: 5*′*-CTCCTAGGCATCCAACCAAA-3*′ *REV: 5*′*- GTGGCTCCCAAGTACTGGTC-3*′	373
*DRD 2-10 (rs67800399 merged into rs2734832)*	*FOR: 5*′*- TCAGGTCATTTTGGAAGTTGC-3*′ *REV: 5*′*-AGGGAAGGGGTTGTTGAAAG-3*′	249
*DRD 2-11 (rs1962262)*	*FOR: 5*′*-CCTCAGCCTCCCAAGTATCT-3*′ *REV: 5*′*-TCTTGGTAACCCTGGGAGTC-3*′	240
*DRD 2-12 (rs7131465)*	*FOR: 5*′*-GCCTGTAATCCCAGCACTCT-3*′ *REV: 5*′*-AAGGGAAAACATGGCAAATG-3*′	366
*DRD 2-15 (rs61902807)*	*FOR: 5*′*-CCTCTAAGCACCAGACAGAGC-3*′ *REV: 5*′*-ACCTCAAGAGCCACCGAAA-3*′	250
*DRD 2-16 (rs10789943)*	*FOR: 5*′*-TAGCCTCCTCGCCACTTAGA-3*′ *REV: 5*′*-CGAAAGTTCAGGACCAAGGA-3*′	362
*DRD 2-17 (rs10789944)*	*FOR: 5*′*-TAGCCTCCTCGCCACTTAGA-3*′ *REV: 5*′*-CTCTCCCCCATCCTTAGCTT-3*′	300

*DRD, Dopamine Receptor; *FOR*, forward primer; *REV*, reverse primer; bp, base-pair.*

### Restriction Fragment Length Polymorphism

All of the endonucleases used were purchased from Thermo Scientific (Carlo Erba reagents, Cornaredo, Italy) except for the enzyme *Cac*8I, which was purchased from New England Biolabs (Ipswich, MA, United States). Then, 10 μl of amplified obtained from the PCR reaction was used for enzymatic cutting. The digestion mix was prepared using 2 μl of specific digestion buffer and 1 U of the enzyme in a total volume of 20 μl. The reaction was carried out for 1 h in a thermostatic bath by varying the temperature depending on the enzyme used, as specified in Supplementary Table 1.

The information about each polymorphism is obtainable from the NCBI database; db SNPs with the relative expected digestion fragments predicted by the REBsite software are described in detail in the Supplementary Material.

**FIGURE 2:**
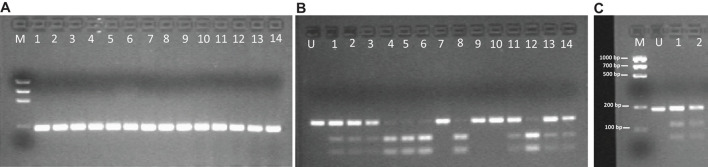
Gel Electrophoresis pattern about *DRD 1-B* SNP (*rs4532*). **(A)** Electrophoretic gel of 14 patients *DRD 1-B* amplicons (187 bp). **(B)** *Bpu*10I enzyme digestion pattern on the same 14 patients PCR amplicons. Restriction site is on T allele. Single 187-bp electrophoresis band denotes a C/C homozygous genotype; 116-bp and 71-bp banding pattern is for a T/T homozygous genotype; the presence of three bands of 187-bp, 116-bp and 71-bp is for C/T heterozygous genotype. **(C)** Molecular length (bp) of restriction fragments derived from *rs4532 digestion by Bpu*10I. M, DNA Molecular Weight Marker (Precision Molecular Mass Ruler, Bio-Rad Laboratories, Inc.); U, undigested PCR amplicon (187 bp).

An example of genotyping, regarding *DRD1-B* (*rs4532*) polymorphism, is shown in Figure 2.

### Statistical Analyses

To determine the relationship between *DRD* SNPs under study and the risk of childhood ADHD, ASD and ASD/ADHD overlap phenotypes, both genotypic and allelic frequencies related to each SNP were compared among the groups reported above and the group of subjects unaffected by any neuropsychiatric pathology (control group) by the Chi-squared test or the Fisher’s Exact test, where appropriate, (empirical *P*-value).

Further the genotypic association analysis under the dominant and recessive models of inheritance were performed.

The differences were considered statistically significant if the *P*-value was < 0.05.

For the latter, the odds ratio (OR) and the 95% confidence interval (95% CI) were then calculated to assess the risk of expressing or not expressing the pathological phenotype for the group under examination compared to the reference group, based on the presence of the minor allele.

A multiple testing correction (false discovery rate) was performed to guard against the potential for false positive associations (corrected *P*-value). Data were analyzed with R version 4.0.2.

## Results

We recruited 75 patients with ASD, 75 patients with ADHD and 30 patients with ASD/ADHD overlap. Demographic features are summarized in Table 2, while allele frequencies distribution, regarding 18 analyzed SNPs, is shown in Table 3.

**TABLE 2:** Demographic features of the study groups.

Participants		ADHD	ASD	ADHD/ASD overlap	Controls
Number		75	75	30	75
Average age, (years)		10.36	10.57	11.57	12.23
Gender	Male (%)	83	85	76.6	75
	Female (%)	17	15	23.4	25

*ADHD, attention deficit/hyperactivity disorder; ASD, autism spectrum disorder.*

**TABLE 3:** Allele frequencies distribution of SNPs in study groups.

Gene	db SNP (rsID)	Minor/major allele	Minor allele frequency			
			ADHD (*n* = 75)	ASD (*n* = 75)	Overlap (*n* = 30)	Controls (*n* = 75)
*DRD 1*	*DRD 1-A (rs686)*	*G/A*	0.346	0.386	0.383	0.293
	*DRD 1-B (rs4532)*	*C/T*	0.346	0.420	0.383	0.286
	*DRD 1-C (rs265973)*	*T/C*	0.427	0.467	0.433	0.460
	*DRD 1-D (rs265975)*	*T/C*	0.327	0.346	0.317	0.427
	*DRD 2-A (rs1076560)*	*A/C*	0.14	0.14	0.150	0.16
	*DRD 2-B (rs1800497)*	*T/C*	0.17	0.16	0.150	0.21
	*DRD 2-C (rs1079597)*	*A/G*	0.11	0.12	0.150	0.16
	*DRD 2-E (rs7118900)*	*A/G*	0.14	0.17	0.167	0.21
	*DRD 2-1 (rs144851051)*	*T/C*	*0.1*	*0.05*	*0.050*	*0.04*
	*DRD 2-2 (rs11608185)*	*C/T*	*0.66*	*0.67*	*0.500*	*0.61*
*DRD 2*	*DRD 2-7 (rs35352421)*	*T/G*	*0.93*	*0.93*	*0.950*	*0.96*
	*DRD 2-8 (rs2245805)*	*A/C*	*0.23*	*0.21*	*0.350*	*0.23*
	*DRD 2-10 (rs67800399 merged into rs2734832)*	*C/A*	*0.34*	*0.33*	*0.500*	*0.39*
	*DRD 2-11 (rs1962262)*	*T/C*	0.11	0.12	0.176	0.16
	*DRD 2-12 (rs7131465)*	*C/A*	0.33	0.34	0.567	0.43
	*DRD 2-15 (rs61902807)*	*C/T*	0.40	0.45	0.333	0.47
	*DRD 2-16 (rs10789943)*	*A/G*	0.15	0.19	0.133	0.13
	*DRD 2-17 (rs10789944)*	*A/C*	0.16	0.19	0.133	0.14

*SNP, single nucleotide polymorphism; ADHD: attention deficit/hyperactivity disorder; ASD, autism spectrum disorder.*

Among *D1* and *D2* receptor genes, Chi-squared test identified six and seven SNPs, respectively, in genotypic and allelic distribution, characterized by a statistically significant difference both in the case–control comparison and between the pathological groups, with empirical *P*-values < 0.05 (Tables 4, 5).

**TABLE 4:** Results of the comparative analysis of genotype distribution of the SNPs among the study groups.

Polymorphism (rsID)	Compared groups	Empirical *P*-value*	Corrected *P*-value for false discovery rate
*DRD 2-8 (rs2245805)*	Overlap vs. ASD	0.05	0.20
*DRD 2-10 (rs2734832)*	Overlap vs. ASD	0.04	0.15
	Overlap vs. ADHD	0.05	0.15
*DRD 2-12 (rs7131465)*	Overlap vs. ASD	0.005	0.015
	Overlap vs. ADHD	0.003	0.015
*DRD 1-B (rs4532)*	ASD vs. CTR	0.04	0.12
*DRD 1-D (rs265975)*	ADHD vs. CTR	0.04	0.12
*DRD 2-2 (rs11608185)*	Overlap vs. ADHD	0.05	0.15

*Empirical *P*-value: result of Chi-squared test.

*SNPs, single nucleotide polymorphisms; ADHD, attention deficit/hyperactivity disorder; ASD, autism spectrum disorder.*

**TABLE 5:** Results of the comparative analysis of allelic distribution of the SNPs among the study groups with the corresponding OR values.

Polymorphism (rsID)	Compared groups	Empirical *P*-value*	Corrected *P*-value for false discovery rate	OR (95% CI)
*DRD 2-1 (rs144851051)*	ADHD vs. CTR	0.04	0.21	2.6 (1.0054–7.073)
*DRD 2-8 (rs2245805)*	Overlap vs. CTR	0.04	0.08	1.9 (1.0317–3.7817)
	Overlap vs. ASD	0.02	0.08	2.1 (1.1095–4.1077)
	Overlap vs. ADHD	0.04	0.08	1.9 (1.0317–3.7817)
*DRD 2-10 (rs2734832)*	ASD vs. Overlap	0.02	0.09	2.1 (1.1196–3.7949)
	ADHD vs. Overlap	0.03	0.09	1.9 (1.0564–3.5671)
*DRD 2-12 (rs7131465)*	Overlap vs. CTR	0.04	0.32	1.8 (1.0255–3.451)
	Overlap vs. ASD	0.001	0.003	2.7 (1.4701–5.024)
	Overlap vs. ADHD	0.001	0.003	2.8 (1.5581–5.3447)
*DRD 1-B (rs4532)*	ASD vs. CTR	0.02	0.12	1.8 (1.115–2.912)
*DRD 2-2 (rs11608185)*	Overlap vs. ASD	0.02	0.09	0.5 (0.2718–0.9197)
	Overlap vs. ADHD	0.03	0.09	0.5 (0.2803–0.9467)
*DRD 2-15 (rs61902807)*	Overlap vs. CTR	0.04	0.24	0.5 (0.2742–0.9695)

*Empirical *P*-value: result of Chi-squared test or Fisher’s Exact test.

*SNPs, single nucleotide polymorphisms; OR, odds ratio; ADHD, attention deficit/hyperactivity disorder; ASD, autism spectrum disorder.*

About *D1* receptor polymorphisms, the SNP *rs4532* appeared to be associated with a greater risk for ASD (OR = 1.8; 95%IC = 1.115–2.912; empirical *P*-value = 0.02).

The most relevant results came from the analysis of D2 receptor polymorphisms. Indeed, *rs2245805* and *rs7131465* appeared to be associated with the increased risk of developing ASD/ADHD overlap compared to the other clinical phenotypes.

The presence of the minor allele in *rs144851051* and *rs2734832* seems to promote the development of a singular clinical disease, that is ADHD vs. controls for *rs144851051* (OR = 2.6; 95%IC = 1.0054–7.073; empirical *P*-value = 0.04) and ADHD or ASD vs. overlap for *rs2734832* (OR = 1.9; 95%IC = 1.0564–3.5671; empirical *P*-value = 0.03 and OR = 2.1; 95%IC = 1.1196–3.7949; empirical *P*-value = 0.02, respectively).

By contrast, *rs11608185* and *rs61902807* could be protective factors for the development of the overlap condition.

However, the false discovery rate method dramatically reduced the number of significantly different SNPs and only *rs7131465* (*DRD2-12*), both in genotypic and allelic distribution, remained after the correction.

The presence of the minor allele in SNP *rs7131465*, located in the 5′-terminal untranslated region (5′ UTR) of the *DRD2* gene, seems to be a strong risk factor of developing ASD/ADHD overlap vs. a singular clinical disease, that is ASD or ADHD (OR = 2.7; 95%CI = 1.4701–5.024; corrected *P*-value = 0.003 and OR = 2.8; 95%CI = 1.5581–5.3447; corrected *P*-value = 0.003, respectively).

Actual results are impacted by the reduced sample size of overlap group and low statistical power of the comparison groups. To solve a similar situation, Ma et al. (2021) merged their data to form a single aggregated clinical group to be compared against a single aggregated control one. The advantage of this method is enlarging the comparison group size and thus increasing statistical power.

On our side, an enlarged ADHD or ASD group, including ADHD/ASD overlap, would lead to the loss of distinctive feature and prediction of specific risk for overlap patients.

To support our findings about rs7131465 SNP, we took a different approach that is dominant and recessive models of inheritance (Table 6; Liu et al., 2021; Ma et al., 2021).

**TABLE 6:** The genotype distribution of SNP *DRD 2-12* between overlap and the other clinical groups and risk prediction for overlap disorder, under the most significant genetic model of inheritance.

db *SNP* (rsID)	Compared groups	Most significant model	Genotype	Group1/Group2 (n, %)	^#^OR (95% CI)	Empirical *P*-value	Corrected *P*-value
*DRD 2-12 (rs7131465)*	Overlap vs. Controls	Dominant	C*/C* + A/C*	26 (87)/50 (67)	3.25 (1.11–11.91)	0.04	0.08
			A/A	4 (13)/25 (33)			
	Overlap vs. ASD	Dominant	C*/C* + A/C*	26 (87)/43 (57)	4.84 (1.68–17.61)	0.007	**0.021**
			A/A	4 (13)/32 (43)			
	Overlap vs. ADHD	Dominant	C*/C* + A/C*	26 (87)/40 (53)	5.69 (1.98 –20.69)	0.003	**0.017**
			A/A	4 (13)/35 (47)			

*OR, odds ratio; SNP, single nucleotide polymorphism; CI, confidence interval.*

*Minor allele.

*Dominant model: homozygous minor allele plus heterozygous vs. homozygous major allele.*

*^#^OR value associated with the minor allele genotype.*

*Significant SNPs after multiple testing correction bolded.*

The group with the C^∗^/C^∗^ homozygous minor allele or the A/C^∗^ heterozygous genotypes of *rs7131465* showed an increased risk of overlap comparing to healthy controls (OR = 3.25, 95% CI = 1.11–11.91, empirical *P*-value = 0.04), ASD (OR = 4.84, 95% CI = 1.68–17.61, empirical *P*-value = 0.007) or ADHD (OR = 5.69, 95% CI = 1.198–20.69, empirical *P*-value = 0.003) in a dominant model, but not a recessive model. The last two of them survived the multiple testing correction and remained statistically significant, i.e., overlap vs. ASD (corrected *P*-value = 0.021), overlap vs. ADHD (corrected *P*-value = 0.017).

No significant difference in the genotype distribution of *rs7131465* in children with ASD or ADHD and healthy controls was observed, both in recessive or dominant model.

## Discussion

In this study, we aimed to investigate if specific *DRD1* and *DRD2* receptor polymorphisms might be considered as potential genetic risk factors for ASD, ADHD, and ASD/ADHD overlap.

Our study found that two specific polymorphisms of the *D2* receptor, *rs2245805* and *rs7131465*, respectively, *DRD2-8* and *DRD2-12*, might be associated with ASD/ADHD overlap when compared with ASD, ADHD, and control groups. However, only the SNP *rs7131465* (*DRD-12*) showed a statistically significant higher risk for the ASD/ADHD overlap.

*DRD2-12* is within the intronic region between exon 1 and exon 2 of 5′ UTR. This intronic region is large 50,391 base pair and *rs7131465* is located near the beginning of exon 2. Currently, no study has been conducted that examined the effect of this polymorphism. Since UTRs are the regulatory elements of genes, acting as controllers of translation and RNA decay, as well as targets for RNA interference (RNAi) and playing a central role in post-transcriptional regulation, it should be no surprise that polymorphisms in 5′ UTRs have been linked to many human, mainly oncological and neurological, diseases (Halvorsen et al., 2010). These SNPs can promote tumorigenesis by increasing c-Myc expression (Chappell et al., 2000), translation inhibition (Cazzola and Skoda, 2000), and transcription activity (Fan et al., 2013). 5′ UTR alterations was also involved in neurological disease such as spinocerebellar ataxia type 1 (Rachna et al., 2020), Parkinson’s disease (Rubino et al., 2020), bipolar disorder type I (Alizadeh et al., 2019) and Alzheimer’s disease (Lahiri et al., 2003).

To the best of our knowledge, no previous studies were carried out to investigate genetic polymorphisms of ASD/ADHD overlap. This is probably related to the fact that the nosographical recognition of the comorbidity between the two disorders occurred only after the publication of the fifth edition of the Diagnostic and Statistical Manual of Mental Disorders in 2013.

However, from the results of the present study, it is hypothesized that dopaminergic neuromodulation may also be involved in the pathogenesis of the overlap, probably with different genetic risk aspects compared to those of ASD and ADHD. Even if these two disorders share a common clinical ground, including the impairment in cognitive functions (e.g., attention skills), in social abilities, and in the executive functions, recent studies underlined that both ASD and ADHD retain qualitative and quantitative clinical differences in their phenotype (Craig et al., 2015; Antshel and Russo, 2019). SNP *rs7131465* in 5′ UTR might be involved in alternative splicing resulting in mRNA instability and producing different isoforms of *DRD2* transcript.

Other *D1* and *D2* receptors have been previously identified in patients with ASD (Hettinger et al., 2008, 2012). A study on murine models showed that excessive striatal dopaminergic activation, deriving from specific mutations of the *D1* receptor, might promote autistic symptoms in mice, such as social deficits and repetitive behaviors. This interpretation was supported by the evidence that murine behavioral changes induced by excessive dopaminergic activity were inhibited by specific *D1* receptor antagonists (Lee et al., 2018).

Interestingly, Liu et al. (2020) demonstrated that certain SNPs of dopaminergic system genes might have a modulator effect on facial/emotion recognition in patients with ASD (Liu et al., 2020). Nevertheless, a recent Chinese study showed that some serotonin HTR2A receptor SNP might also be associated with a higher risk for ASD (Liu et al., 2021). Moreover, previous meta-analyses showed a significant association between some *D2* receptor polymorphisms and ADHD (Sullivan et al., 2012; Wu et al., 2012; Pan et al., 2015). As for ASD, some studies investigated the possible effects of gene polymorphisms of the DS on the functional activity of the dopaminergic circuits involved in ADHD. Different models have been proposed to explain the symptomatology of the disorder; among these, the executive functions model is the most described and studied (Arnsten and Li, 2005; Willcutt et al., 2005; Craig et al., 2016).

Lastly, more recent neuroimaging studies showed that the presence of some *DRD2* and *DRD4* might, respectively, modulate the gyrification and the functional activity of cortical areas involved in cognitive processes that are impaired in ADHD and other psychiatric disorders (Palaniyappan et al., 2019; Overs et al., 2021).

## Conclusion

In conclusion, we found that carrying specific *DRD1/DRD2* SNPs could increase the risk for ASD, ADHD, even if only one SNP showed a statistically significant association with a higher risk for and ASD/ADHD overlap. These findings might support the hypothesis of the involvement of the dopaminergic system in the neurobiology of these conditions. However, this study has some limitations that need to be mentioned. The study protocol approved by the Local Committee did not include also genetic examination of the parents’ patient; therefore, we were not able to verify if a SNP was inherited or it is a *de novo* mutation. Moreover, this was a genetic preliminary study, so we did not proceed with a functional validation of the analyzed SNPs and with a correlation phenotype/genotype analysis; however, all these analyses would be considered for future investigations.

In addition, further studies on larger groups might explore more in-depth how the dopaminergic system SNPs could represent biomarkers for a clinical phenotype and eventually how they could modulate the efficacy of the pharmacological or rehabilitation therapy in these disorders.

## Data Availability Statement

The datasets presented in this study can be found in online repositories. The names of the repository/repositories and accession number(s) can be found in the article/Supplementary Material.

## Ethics Statement

The studies involving human participants were reviewed and approved by Local Ethical Committee—Policlinico of Bari (protocol number 592/12). Written informed consent to participate in this study was provided by the participants’ legal guardian/next of kin.

## Author Contributions

MM, RP, and LM: conceptualization, writing—review and editing, supervision, and project administration. MM, RP, AnV, AlV, and RL: methodology and data curation. AnV, AlV, and RL: formal analysis. MM, RP, MP, AP, AG, OG, and RL: investigation. AnV, MP, AP, AG, and OG: resources. MM and RP: writing—original draft preparation. All authors contributed to the article and approved the submitted version.

## Conflict of Interest

The authors declare that the research was conducted in the absence of any commercial or financial relationships that could be construed as a potential conflict of interest.

## Publisher’s Note

All claims expressed in this article are solely those of the authors and do not necessarily represent those of their affiliated organizations, or those of the publisher, the editors and the reviewers. Any product that may be evaluated in this article, or claim that may be made by its manufacturer, is not guaranteed or endorsed by the publisher.

## Footnotes

1
https://www.neb.com/

2
http://tools.neb.com/NEBcutter2/

3
http://tools.neb.com/REBsites/index.php
