# Supplementary material for: DRD1 and DRD2 Receptor Polymorphisms: Genetic Neuromodulation of the Dopaminergic System as a Risk Factor for ASD, ADHD and ASD/ADHD Overlap
Source: Front Neurosci. 2021 Sep 29;15:705890. doi: 10.3389/fnins.2021.705890 (PMC8511701; doi:10.3389/fnins.2021.705890)
Supplement: Supplementary file 1 [file Data_Sheet_1.PDF]

Supplementary Material

Information about each polymorphism obtained from the NCBI database (dbSNP) and the relative expected digestion fragments predicted by the REBsite software are reported below. Global minor allele frequency (MAF) is reported for each rs included in the global population based on 1000Genome phase 3 genotype data. The minor allele was considered to be the polimorphic allele.

rs686 A/G (DRD1-A)

The *rs686* A/G (forward, FWD) polymorphism is located in the 3' UTR of the *DRD1* gene and it has a good distribution in the population (dbSNP). The MAF for the minor allele "G" is equal to 0.395 that is the polymorphic *rs686* "G" allele is present in 39.5% of the population (dbSNP). To discriminate the *rs686* "A" allele from the *rs686* "G" allele, the *Cac8I* enzyme that able to cut the polymorphic sequence in *rs686* "G" allele was used. By adding information about the amplified sequence and the endonuclease used, the REBsite program predicted the formation of two bands at the end of the digestion, one of 95 bp (base pairs) and the other one of 71 bp.

In the case of patients homozygous for the *rs686* "A" allele, you will see a single band of 166 bp on the agarose gel (the enzyme does not recognize the polymorphic sequence, therefore, there is no cutting); in the case of patients homozygous for the *rs686* "G", the 95bp band and the 71bp band will be present; in contrast, for patients with the heterozygous "AG" genotype, three bands (of 166, 95 and 71 bp each one) will be seen.

rs4532 C/T (DRD1-B)

The SNP *rs4532* C/T (FWD) is located in the 5' UTR of the *DRD1* gene. It is less widespread than the previous one, in fact the MAF for the polymorphic *rs4532* "C" allele is 0.245 (dbSNP). The enzyme *Bpu10I* used recognizes the *rs4532* "T" allele so that its cutting on the amplified sequence will generate two restriction fragments, one of 116 bp and the other one of 71 bp. Therefore, we expect to observe the following electrophoretic patterns depending on the patient genotype: two electrophoretic bands of 116 bp and 71 bp each one in case of the homozygous "TT" genotype; a single band of 187 bp in case of the homozygous "CC" genotype; three bands of 187, 116 and 71 bp each one in case of the heterozygous "TC" genotype.

rs265973 C/T (DRD1-C)

The *rs265973* C/T (FWD) polymorphism maps on chromosome 5 near the *DRD1* gene. The *rs265973* "T" allele (MAF = 0.455) and *rs265973* "C" allele have a similar allelic frequency (dbSNP), therefore it is not clear which allele is to be considered as polymorphic allele. We considered the *rs265973* "T" minor allele as the polymorphic one. The enzyme used in this case, *RsaI*, recognizes the *rs265973* "C" allele whose cutting on the amplified generates two bands visible on the agarose gel, one of 71 bp and the other one of 29 bp.

Depending on the patient genotype, there will be the following electrophoretic patterns: for the homozygous "CC" status there will be two bands of 71 and of 29 bp each one; for the homozygous

"TT" genotype a single band of 100 bp; for the heterozygous "CT" genotype three bands of 100 bp, 71 bp and 29 bp each one.

rs265975 C/T (DRD1-D)

The *rs265975* C/T (FWD) polymorphism, like the previous polymorphism, is located in a region near the gene coding for the *DRD1* receptor. This polymorphism is also widespread in the population; in fact, the *rs265975 "T"* allele has a MAF of 0.436. The *HaeIII* endonuclease recognizes and cuts the *rs265975* "C" allele leading to the formation of 79 and 153 bp bands.

The following are the expected electrophoretic patterns: for the homozygous "CC" genotype, two bands of 79 bp and 153 bp respectively will be visible; for the homozygous "TT" genotype a single band of 232 bp will be visible; for the heterozygous "CT" genotpe three bands of 232 bp, 153 bp, and 79 bp each one will be visible.

rs1076560 A/C (DRD2-A)

The *rs1076560* A/C (FWD) polymorphism is located in the promoter region of the *DRD2* gene. The MAF for the polymorphic *rs1076560* "A" allele is 0.230. The enzyme used in this case, *HphI,* recognizes *rs1076560* "A" allele and the digestion performed by this enzyme leads to the formation of three fragments of 34 bp, 39 bp, and 139 bp each one. The first two fragments will coalesce on the agarose gels because they have a very similar molecular weight.

The following electrophoretic patterns will be obtained: for the homozygous "CC" genotype we will see one fragment of 139 bp and the other fragment of 73 bp resulting from the union of the 34 bp band with the 39 bp band; for the homozygous "AA" genotype we will see two fragments, one of 139 bp and the other one of 34/39 bp that are the fragments migrating together; for the heterozygous "AC" status, you will see three fragments: one of 139 bp, one of 73 bp and last one of 34/39 bp.

rs1800497 C/T (DRD2-B)

The *rs1800497* C/T (reverse, REV) polymorphism is located on chromosome 11, it maps close to the *DRD2* gene promoter within the A*nkyrin* gene. The MAF of the *rs1800497* "T" polymorphic allele is equal to 0.326 whose presence has been attributed to a pathogenetic role. The restriction enzyme *TaqI* recognizes and cuts the wild type allele generating two fragments of 159 bp and 134 bp each one.

The expected electrophoretic patterns are the following: for the homozygous "CC" genotype, two bands, one of 134 bp and the other one of 159 bp; for the homozygous "TT"genotype, a single band of 293 bp; for the heterozygous "CT" genotype, three bands respectively of 293 bp, 134 bp, and 159 bp.

rs1079597 A/G (DRD2-C)

The *rs1079597* A/G (REV) polymorphism is located in an intronic region of the *DRD2* gene. The MAF of *rs1079597* "A" polymorphic allele is 0.251. The endonuclease used to discriminate the two allelic variants is the *TaqI* enzyme which digests the wild type allele rs1079597 "G", generating two fragments of 169 bp and 104 bp each one. The electrophoretic patterns obtained are the following: for the homozygous "GG" genotype, a bands of 169 bp and another band of 104 bp; for the

homozygous "AA" genotype, a single electrophoretic 273 bp band will be visible; for the heterozygous "GA" there will be three bands respectively of 273 bp, 169 bp, and 104 bp.

rs7118900 A/G (DRD2-E)

The *rs7118900* A/G (FWD) polymorphism is located in the A*nkyrin* gene (*ANKK1*). The MAF of the *rs7118900* "A" polymorphic allele is 0.320 and its presence involves the aminoacid substitution of Alanine with the Threonine. The restriction enzyme used, *Tsp45I*, recognizes the *rs7118900* "A" allele leading to the formation of two fragments, one of 224 bp and the other one of 124 bp.

The following are the expected electrophoretic patterns: for the homozygous "GG"genotype, a single band of 348 bp; for the homozygous "AA"genotype, two bands of 224 bp and 124 bp; for the heterozygous "GA" genotype, three bands respectively of 348, 224 and 124 bp.

rs144851051 C/T (DRD2-1)

The *rs144851051* C/T (FWD) polymorphism is located in an intronic region belonging to a CpG island region of the *DRD2* gene and it is uncommon in the population. The MAF of the *rs144851051* "T" polymorphic variant is 0.085. The restriction enzyme used *TaqI* recognizes the wild type sequence generating two fragments of 223 bp and 123 bp.

Depending on the patient genotype, we will have the following electrophoretic patterns: for the homozygous "CC" genotype, two bands, one of 223 bp and the other one of 123 bp; for the homozygous "TT"genotype, a single band of 346 bp; for the heterozygous "CT" genotype, three bands of 346 bp, 223 bp and 123 bp each one.

rs11608185 T/C (DRD2-2)

The SNP *rs11608185* T/C (FWD) is located in an intronic region within a CpG island region. The MAF of the *rs11608185* "C" allele is 0.294. The used endonuclease, *MspI*, digests the amplified sequence in the polymorphic site leading to the formation of two bands of 236 bp and 160 bp each one. The expected electrophoretic patterns are the following: for the homozygous "TT"genotype, a single band of 396 bp; for the homozygous "CC" genotype, two bands of 236 bp and 160 bp; for the heterozygous "TC" genotype, three bands of 396 bp, 236 bp, and 160 bp each one.

rs35352421 G/T (DRD2-7)

For the *rs35352421* G/T (FWD) polymorphism, the MAF of the polimorphic allele "T" is 0.027. The cutting by the enzyme *EarI* produces four restriction fragments of 131 bp, 83 bp, 214 bp and 161 bp each one, of which the last two co-migrate together on the agarose gel. The following are the expected electrophoretic patterns: for the homozygous "GG" genotype, two closed bands of 214 and 161 bp; for the homozygous "TT" genotype, three bands of 131bp, 83 bp and 161 bp each one; for the heterozygous "GT" genotyape, four bands of 131 bp, 83 bp, 214 bp and 161 bp each one.

rs2245805 A/C (DRD2-8)

The *rs2245805* A/C (REV) polymorphism has the same location as the previous polymorphisms and it is quite widespread in the population; in fact, the frequency of the less represented *rs2245805* "A" variant is 0.420. In addition to the *rs2245805* polymorphic site "A", the enzyme *BseNI* recognizes two other sites. The cutting of the enzyme on the amplified sequence produces 4 restriction

fragments of 192 bp, 109 bp, 53 bp, and 19 bp each one, of which these last two tend to migrate together on the agarose gel. There will be the following electrophoretic patterns: for the homozygous "CC" genotype, two bands, one of 301 bp and the other one of 59 bp (together with whom the fragment of 19 bp migrates); for homozygous "AA"genotype, three bands of 192 bp, 109 bp and 19-53 bp each one; for the heterozygous "CA" genotype, four bands of 301 bp, 192 bp, 109 bp, 19-53 bp each one.

rs67800399 now merged into rs2734832 C/T (DRD2-10)

About the *rs67800399* C/T (REV) polymorphism, the MAF of the *rs67800399* "C" polymorphic allele is 0.278. The enzyme *MspI* recognizes the wild type sequence *rs67800399* "C" producing three fragments of 120 bp, 83 bp and 46 bp each one, because there is also an additional site in the amplified sequence, that is recognized by *MspI*.

The expected electrophoretic patterns are the folliwing: for the homozygous "GG" genotype, three bands of 120 bp, 83 bp, and 46 bp each one; for the homozygous "AA"genotype, two bands of 166 bp and 83 bp each one; for the heterozygous "AG" genotype, four bands of 166 bp, 120 bp, 83 bp, and 46 bp each one.

rs1962262 C/T (DRD2-11)

For the *rs1962262* C/T (REV) polymorphism, the enzyme used was *MseI*; it recognizes and cuts the polymorphic site "T", resulting in two electrophoretic bands of 172 bp and 68bp each one.

The expected electrophoretic patterns after the restriction are the following: for the homozygous "CC"genotype, a single 240 bp band; for the homozygous "TT", two bands of 172 bp and 68 bp each one; for the C>T heterozygous "CT"genotype, three bands of 240 bp, 68 bp and 172 bp each one.

rs7131465 A/C (DRD2-12)

The *rs7131465* A/C (FWD) polymorphism is located on the *DRD2* gene, the restriction enzyme *BtgI* recognizes and cuts the polymorphic sequence in the minor allele "C" (MAF=0.289), producing two electrophoretic bands of 225 bp and 141 bp respectively. The expected electrophoretic patterns are the following: for the homozygous "CC" genotype, two bands of 225 bp and 141bp each one; for the homozygous "AA" genotype, a single band of 366 bp; for the heterozygous "CA" genotype, three band of 366 bp, 225 bp and 141 bp each one.

rs61902807 T/C (DRD2-15)

For the *rs61902807* T/C (FWD) polymorphism, the MAF for the allele "C" is 0.183. The restriction enzyme *NlaIII* recognizes and cuts the wild type "T" site, resulting in a band of 132 bp and another band of 118 bp. The expected electrophoretic patterns are the following: for the homozygous "TT" genotype, two bands of 132 bp and 118 bp each one; for the homozygous "CC" genotype, a single band of 250 bp; for the heterozygous "TC" genotype, three bands of 250 bp, 132 bp, and 118 bp each one.

rs10789943 G/A (DRD2-16)

For the *rs10789943* G/A (FWD) polymorphism, the MAF for the allele "A" is 0.211. The restriction enzyme *Tsp509I* cuts in the presence of the polymorphic "A" site , producing two bands of 239 bp

and 123 bp respectively. The following are the expected electrophoretic patterns: for the homozygous “AA” genotype, two bands of 239 bp and 123 bp each one; for the homozygous “GG” genotype, a single band of 362 bp; for heterozygous “GA” genotype, three bands of 239 bp, 123 bp, and 362 bp respectively.

rs10789944 C/A (DRD2-17)

For the *rs10789944* C/A (FWD) polymorphism, the MAF for the allele “A” is 0.218. The restriction enzyme *TaqI* cuts the *rs10789944* “A” polymorphic site producing two bands of 178 bp and 127 bp each one. The following are the expected electrophoretic profiles: for the homozygous “CC”genotype, a single band of 300 bp; for the homozygous “AA”genotype, two bands of 178 bp and 127 bp each one; for the heterozygous “CA” genotype, three bands of 300 bp, 178 bp, and 127 bp each one.

***Table S1**. List of the restriction enzymes used and their relative incubation temperature.*

Enzyme	*Incubation temperature*
Cac8I	37 °C
Bpu10I	37 °C
RsaI	37 °C
HaeIII	37 °C
HphI	37 °C
TaqI	65 °C
Tsp45I	37 °C
MspI	37 °C
BseNI	65 °C
EarI	37 °C
MseI	65 °C
BtgI	37 °C
NlaIII	37 °C
Tsp509I	65 °C
